# Investigating the causal association between branched-chain amino acids and Alzheimer's disease: A bidirectional Mendelian randomized study

**DOI:** 10.3389/fnut.2023.1103303

**Published:** 2023-03-31

**Authors:** Xiao-hang Qian, Xiao-li Liu, Bin Zhang, Yuan Lin, Jian-hua Xu, Gang-yu Ding, Hui-dong Tang

**Affiliations:** ^1^Department of Geriatrics, Ruijin Hospital, Shanghai Jiao Tong University School of Medicine, Shanghai, China; ^2^Medical Center on Aging of Ruijin Hospital, Shanghai Jiao Tong University School of Medicine, Shanghai, China; ^3^Department of Neurology and Institute of Neurology, Ruijin Hospital, Shanghai Jiao Tong University School of Medicine, Shanghai, China; ^4^Department of Neurology, Shanghai University of Medicine and Health Sciences Affiliated Sixth People's Hospital South Campus, Shanghai, China; ^5^Department of Gastroenterology, Jiading District Central Hospital Affiliated Shanghai University of Medicine and Health Sciences, Shanghai, China; ^6^Department of Neurology, Jiading District Central Hospital Affiliated Shanghai University of Medicine and Health Sciences, Shanghai, China

**Keywords:** Alzheimer's disease, branched-chain amino acids, valine, leucine, isoleucine, Mendelian randomized study

## Abstract

**Background:**

There are many metabolic pathway abnormalities in Alzheimer's disease (AD). Several studies have linked branched-chain amino acid (BCAA) metabolism disorders with AD but have not obtained consistent results. The purpose of this study is to explore the causal association between BCAA concentration and the risk of AD.

**Methods:**

A bidirectional Mendelian randomized (MR) study was applied to explore the causal effect between BCAA level and the risk of AD. Genetic instrumental variables from the genome-wide association study (GWAS) of serum BCAA levels [total BCAAs (115,047 participants), valine (115,048 participants), leucine (115,074 participants), and isoleucine (115,075 participants)] from the UK Biobank and AD (21,982 AD cases and 41,944 controls) from the International Genomics of Alzheimer's Project were applied to explore the causal effect through the inverse variance-weighted (IVW) method, MR-Egger, and weighted median, accompanied by multiple pluripotency and heterogeneity tests.

**Results:**

The forward MR analysis showed that there was no causal effect of total BCAAs (OR: 1.067, 95% CI: 0.838–1.358; *p* = 0.838), valine (OR: 1.106, 95% CI: 0.917–1.333; *p* = 0.292), leucine (OR: 1.096, 95% CI: 0.861–1.396; *p* = 0.659), and isoleucine (OR: 1.457, 95% CI: 1.024–2.742; *p* = 0.037) levels on the risk of AD. The reverse analysis showed that AD was related to reduced levels of total BCAAs (OR: 0.979, 95% CI: 0.989–0.990; *p* < 0.001), valine (OR: 0.977, 95% CI: 0.963–0.991; *p* = 0.001), leucine (OR: 0.983, 95% CI: 0.973–0.994; *p* = 0.002), and isoleucine (OR: 0.982, 95% CI: 0.971–0.992; *p* = 0.001).

**Conclusion:**

We provide robust evidence that AD was associated with a decreased level of BCAAs, which can serve as a marker for early diagnosis of AD.

## Introduction

Alzheimer's disease (AD), the main type of dementia, becomes one of the most serious public health threats in the world (1). It is estimated that there are currently more than 50 million people worldwide with dementia, which is expected to triple in 2050 (2). Among them, AD accounts for 60–80% of dementia (3). Typical pathological features of AD include extracellular amyloid-β (Aβ) plaques accumulated by Aβ and intracellular neurofibrillary tangles formed by phosphorylated tau (4). However, there is still a lack of accurate explanation of the pathogenesis and effective disease-modifying treatment for AD (5). Increased evidence suggests that the disruption of various metabolic pathways is another important feature of AD (6, 7). Metabolites are the end biochemical products of various biological pathways, such as amino acids, peptides, lipids, and nucleic acids (8). They can reflect the alteration in the complex biological pathways involved in AD caused by the interaction of genetic, environmental, and behavioral factors (6).

Branched-chain amino acids (BCAAs), including leucine (Leu), isoleucine (Ile), and valine (Val), are three types of essential amino acids in the human diet (9). They are not only involved in protein synthesis but also possess various metabolic pathways (10, 11). Altered BCAA metabolism has been shown to be associated with AD in an increasing number of studies (12). However, different studies showed controversial and mixed results. For instance, a previous study of the cerebrospinal fluid (CSF) and plasma amino acid composition has demonstrated a significant reduction of valine in AD patients compared to healthy controls (HC) (13). In a study of sporadic AD patients without receiving any medication, alterations of 23 metabolites were detected, including significantly decreased valine levels (14). In addition, a longitudinal study in APP/PS1 transgenic mice involving profiling of the brain and the plasma metabolome has found seriously disturbed polyamines and BCAA metabolism (15). Notably, the plasma levels of valine were shown to be significantly reduced in AD mice. In a more recent study, lower plasma valine level was shown to correlate with the rate of cognitive decline (16). Nonetheless, there were elevated BCAA concentrations in the serum of AD patients in a small sample study (17). Similarly, elevated isoleucine levels have been observed in patients with mild cognitive impairment (MCI) (18). These heterogeneous results may be related to the susceptibility of metabolites influenced by multiple factors, such as lifestyle and diet, immune response, genetic variations, and gut microbiota (19). These uncontrollable confounding factors make it difficult to distinguish symptoms from causes. Therefore, the use of more reliable research methods will help to elucidate the causal association between BCAA levels and the risk of AD.

Mendelian randomization (MR) study is a methodology that can be applied to explore the causal relationships between exposures (risk factors) and outcomes (diseases) by using genetic variants (20). The genetic variants are only related to the risk factors but are not affected by any confounders (21). In this study, a bidirectional Mendelian randomized approach was applied to explore the causal effects between BCAA levels and the risk of AD.

## Methods

### Study design overview

**Figure 1:**
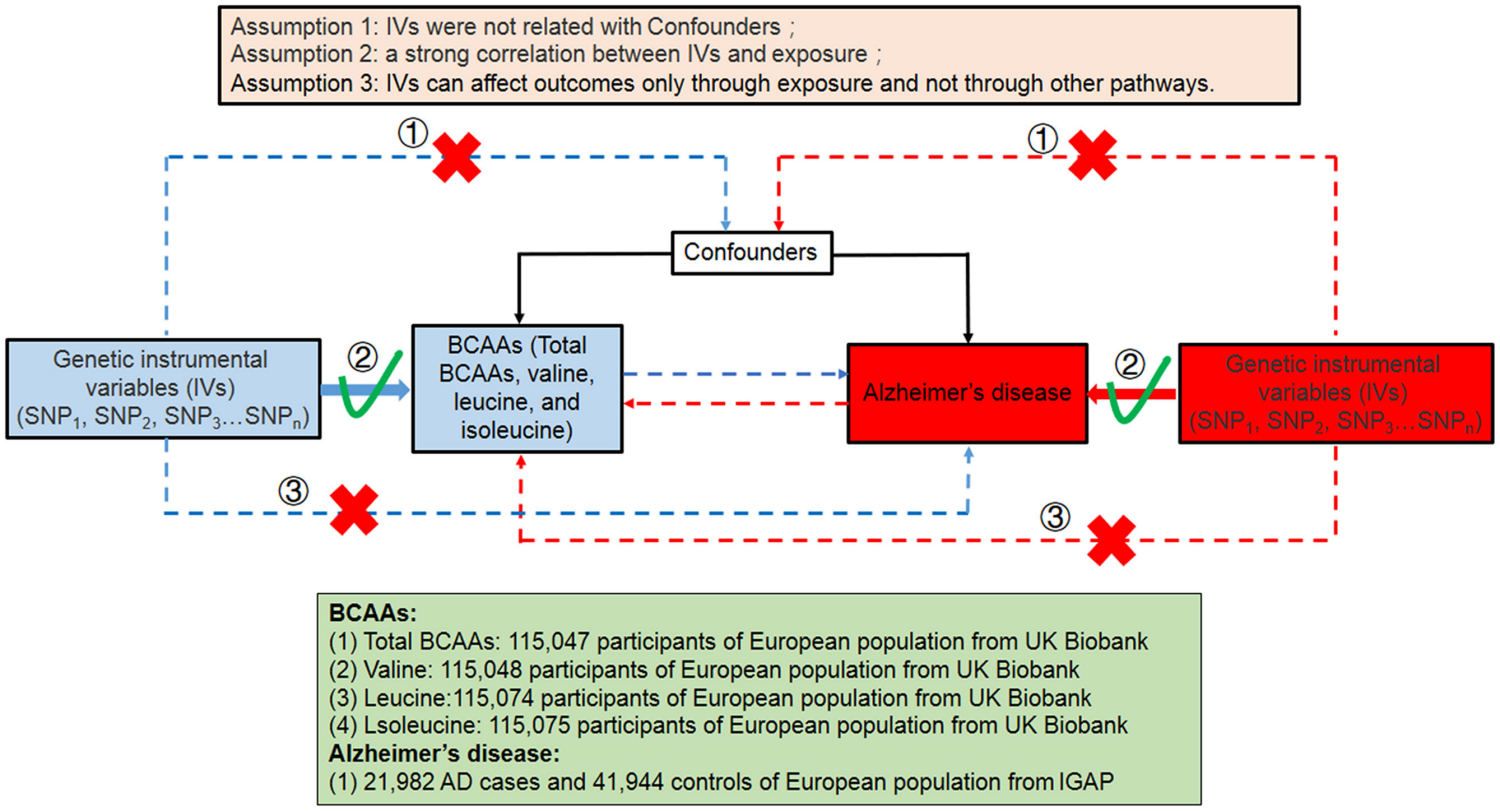
Summary of this bidirectional MR study between the level of BCAAs and the risk of AD in this study. SNP, single-nucleotide polymorphism; BCAAs, branched-chain amino acids; AD, Alzheimer's disease.

We performed a bidirectional Mendelian randomized study to assess the causal effect between BCAA levels and the risk of AD (Figure 1). The bidirectional Mendelian randomization study was built on the following three assumptions: First of all, the instrumental variables (IVs) were not related to the confounders. Second, there was a strong correlation between IVs and exposure. Third, IVs can affect outcomes (AD) only through exposure and not through other pathways. The analysis of this study was based on genome-wide association study (GWAS) data of BCAAs and Alzheimer's disease in the public database. Therefore, ethics approval and consent are not required for this study.

### Data sources and selection of genetic instruments

The genetic summary statistics of AD (21,982 AD cases and 41,944 controls) were accessed from the International Genomics of Alzheimer's Project (IGAP), which was composed of four consortia: Cohorts for Heart and Aging Research in Genomic Epidemiology Consortium (CHARGE), the European Alzheimer's Disease Initiative (EADI), Alzheimer's Disease Genetics Consortium (ADGC), and Genetic and Environmental Risk in AD/Defining Genetic, Polygenic and Environmental Risk for Alzheimer's Disease Consortium (GERAD/PERADES) (22). For BCAAs (total BCAAs, valine, leucine, and isoleucine), the GWAS statistics were obtained from the MRC IEU OpenGWAS project (https://gwas.mrcieu.ac.uk/), including 115,047 participants for total BCAAs (GWAS ID “met-d-Total_BCAA”), 115,048 participants for valine (GWAS ID “met-d-Val”), 115,074 participants for leucine (GWAS ID “met-d-Leu”), and 115,075 participants for isoleucine (GWAS ID “met-d-Ile”) from the UK Biobank. All participants were of European ancestry.

Instrumental variables (IVs) for AD and BCAAs were extracted under the same criteria. To be specific, the GWAS statistical difference threshold of all relevant SNPs from each GWAS was set to *P* < 5 × 10^−8^. The PLINK clumping algorithm was applied to prune for the independence of SNPs in linkage disequilibrium under the threshold of r^2^ < 0.001 in a 10,000 kb window (23). The palindromic SNPs with a minor allele frequency (MAF) of <0.01 were excluded from the aforementioned instrument SNPs. The PhenoScanner GWAS database (http://phenoscanner.medschl.cam.ac.uk) was applied to remove possible confounding of the exposure–outcome associations (24). In this study, gender, age, diabetes, and cardiovascular disease were selected as confounders. The SNPs associated with them were excluded. Furthermore, the F statistic was calculated to assess the strength of the selected genetic variants (25). The SNPs used as IVs in this study are presented in Supplementary Table S1. The F statistic is presented in Supplementary Table S2.

### Mendelian randomized analysis

Two-sample MR was applied to explore the causal effect between BCAA levels and the risk of AD. The inverse variance-weighted (IVW) method was applied to the standard MR analysis. In addition, the MR-Egger and weighted median were also performed to verify the causal effect (21, 26). Mendelian randomization pleiotropy residual sum and outlier (MR-PRESSO) global test was conducted to identify the horizontal pleiotropic of IVs (26). Cochran's Q statistic was applied to assess the heterogeneity of IVW and MR-Egger (27). The MR-Egger intercept test was also used to estimate the potential horizontal pleiotropy of the MR results. In addition, the leave-one-out analysis was also used to eliminate potential pleiotropy by assessing the effects of a single IV on causal effect by removing each IV from the IVW method (28). All analyses in this study were performed through the “two-sample MR” and the “MR-PRESSO” packages in R software (v3.60). As four types of exposures were analyzed, the statistically significant threshold was set as *P* < 0.0125 after the Bonferroni correction.

## Results

### Genetically predicted BCAA levels on the risk of AD

**Figure 2:**
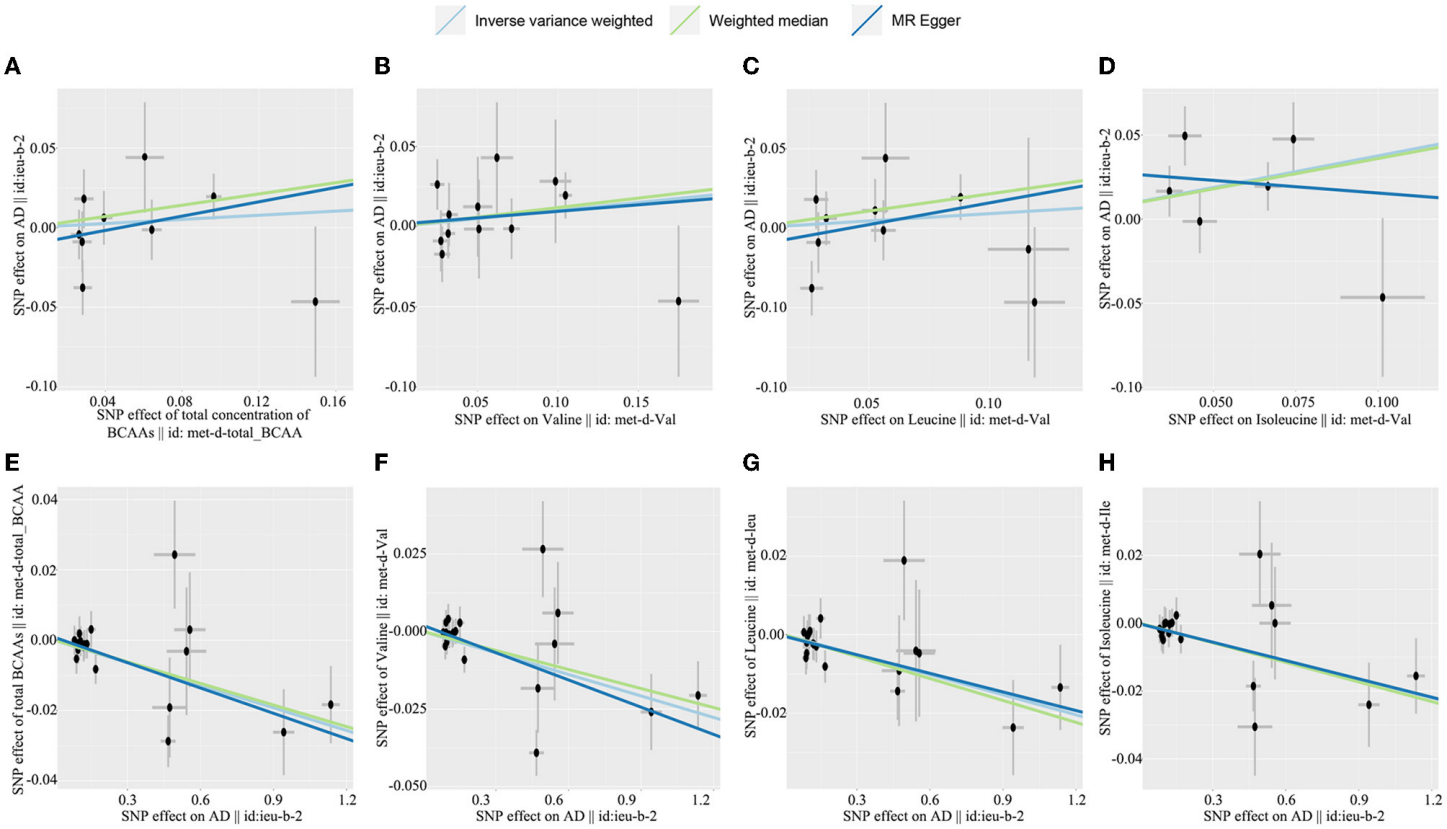
Scatterplot of the genetic causal effect sbetween BCAAs level the risk of AD. **(A)** Total BCAAs on AD, **(B)** valine on AD, **(C)** leucine on AD, **(D)** isoleucine on AD, **(E)** AD on total BCAAs, **(F)** AD on valine, **(G)** AD on leucine, **(H)** AD on isoleucine. BCAAs, branched-chain amino acids; AD, Alzheimer's disease.

The causal effect of BCAA levels on the risk of AD was conducted through the inverse variance-weighted (IVW) MR method. In addition, MR-Egger and weighted median were applied to verify the results of the IVW method. After removing SNPs, the results showed that there were no casual effects on the levels of total BCAAs (IVW, OR: 1.067, 95% CI: 0.838–1.358; *p* = 0.838), valine (IVW, OR: 1.106, 95% CI: 0.917–1.333; *p* = 0.292), leucine (IVW, OR: 1.096, 95% CI: 0.861–1.396; *p* = 0.659), and isoleucine (IVW, OR: 1.457, 95% CI: 1.024–2.742; *p* = 0.037) on the risk of AD (Figures 2A–D, Table 1). Similarly, the results for MR-Egger and weighted median do not suggest an effect of BCAA levels on the risk of AD. Based on these results, we found no causal effect of BCAA levels on the risk of AD.

**Table 1:** Mendelian randomization analysis explore the genetic causal effects of the BCAAs on the risk of AD.

**Exposure**	**Outcome**	**Method**	**No. of SNP**	**MR analysis**				**MR-egger intercept (*p* value)**	**MR-PRESSO global test (*p* value)**	**Cochran's Q (*p* value)**
				β **value**	**OR**	**95% CI**	***P*** **value**			
Total BCAAs	AD	IVW	9	0.065	1.067	0.838–1.358	0.838	−0.011 (0.474)	0.135	10.312 (0.244)
		WM	9	0.177	1.193	0.918–1.550	0.918			
		MR egger	9	0.225	1.252	0.773–2.028	0.772			
Valine	AD	IVW	12	0.100	1.106	0.917–1.333	0.292	0.001 (0.900)	0.451	8.131 (0.701)
		WM	12	0.119	1.126	0.894–1.419	0.315			
		MR egger	12	0.081	1.085	0.767–1.535	0.656			
Leucine	AD	IVW	10	0.092	1.096	0.861–1.396	0.659	−0.011 (0.482)	0.164	10.311 (0.325)
		WM	10	0.216	1.241	0.944–1.634	0.869			
		MR egger	10	0.275	1.316	0.763–2.269	0.352			
Isoleucine	AD	IVW	6	0.376	1.457	1.024–2.742	0.037	0.031 (0.435)	0.199	8.888 (0.113)
		WM	6	0.361	1.434	1.014–2.029	0.414			
		MR egger	6	−0.150	0.860	0.248–2.986	0.824			

IVW, inverse variance-weighted; BCAAs, branched-chain amino acids; AD, Alzheimer's disease; OR, odd ratio; CI, confidence interval.

### Genetically predicted AD on the BCAA levels

In the reverse MR analysis, we explored the causal effect of AD on the BCAA levels through IVW, MR-Egger, and weighted median. Our investigation revealed that AD was related to a decreased level of total BCAAs (IVW, OR: 0.979, 95% CI: 0.989–0.990, *p* < 0.001), valine (IVW, OR: 0.977, 95% CI: 0.963–0.991; *p* = 0.001), leucine (IVW, OR: 0.983, 95% CI: 0.973-−0.994, *p* = 0.002), and isoleucine (IVW, OR: 0.982, 95% CI: 0.971–0.992, *p* = 0.001) (Figures 2E–H, Table 2). Similarly, the results of MR-Egger and weighted median were consistent with the results of the IVW method. Taken together, these results revealed that AD was obviously associated with decreased BCAA levels.

**Table 2:** Mendelian randomization analysis explore the genetic causal effects of AD on the level of BCAAs.

**Exposure**	**Outcome**	**Method**	**No. of SNP**	**MR analysis**				**MR-egger intercept (*p* value)**	**MR-PRESSO global test (*p* value)**	**Cochran's Q (*p* value)**
**AD**	Total BCAAs	IVW	19	−0.021	0.979	0.989–0.990	< 0.001	0.001 (0.632)	0.495	18.464 (0.425)
		WM	19	−0.020	0.980	0.964–0.994	0.008			
		MR Egger	19	−0.024	0.976	0.961–0.991	0.007			
**AD**	Valine	IVW	19	−0.023	0.977	0.963–0.991	0.001	0.002 (0.385)	0.022	31.089 (0.028)
		WM	19	−0.020	0.980	0.964–0.995	0.011			
		MR Egger	19	−0.029	0.971	0.952–0.990	0.009			
**AD**	**L**eucine	IVW	19	−0.017	0.983	0.973–0.994	0.002	−0.0004 (0.816)	0.905	10.835 (0.901)
		WM	19	−0.019	0.982	0.968–0.995	0.010			
		MR Egger	19	−0.016	0.984	0.970–0.999	0.050			
**AD**	**I**soleucine	IVW	19	−0.019	0.982	0.971–0.992	0.001	−0.0001 (0.910)	0.707	12.588 (0.815)
		WM	19	−0.019	0.981	0.966–0.996	0.013			
		MR Egger	19	−0.018	0.982	0.967–0.997	0.031			

IVW, inverse variance-weighted; BCAAs, branched-chain amino acids; AD, Alzheimer's disease; OR, odd ratio; CI, confidence interval.

### Sensitivity analyses

There were no obvious heterogeneities in the causal effect between AD and BCAAs (Tables 1, 2). The intercept of the MR-Egger regression and MR-PRESSO test revealed no obvious horizontal pleiotropy (Tables 1, 2). In addition, the included instrumental variables show apparent symmetry in the funnel plot to exclude the directional pleiotropy (Supplementary Figure S1). Meanwhile, the leave-one-out analysis showed all SNPs contributing to consistent causal estimates (Supplementary Figure S2). The aforementioned analysis proves the reliability of the results of the study.

## Discussion

Alzheimer's disease, the most common type of dementia, currently lacks disease-modifying treatment strategies. To make matters worse, the current understanding of the pathogenesis of AD is limited. As a type of essential amino acid, BCAAs have been proven to be closely associated with AD (3, 29). The alteration of BCAA concentration in AD patients is correlated with disease progression and AD-related pathological features (30, 31). However, a causal association between AD and BCAA has not been established. In this study, we attempt to explore the causal effect between AD and BCAAs (total BCAAs, valine, leucine, and isoleucine) using a bidirectional two-sample MR study. The results do not show evidence that BCAA levels are causally related to the risk of AD. Conversely, the reverse MR analysis suggests that AD is significantly associated with decreased BCAA levels, suggesting that AD is the cause but not the result of changed BCAA levels.

Several previous observational cohort studies have linked BCAAs to AD. An earlier study found that there was reduced levels of leucine and valine in the CSF of patients with AD, but no statistically significant differences in plasma (13). In Alzheimer's Disease Neuroimaging Initiative-1 (ADNI-1) cohort, there was a decreased serum valine level in AD patients compared to HC and stable mild cognitive impairment (sMCI) patients (32). Similarly, serum valine levels were lower in patients with progressive MCI (pMCI) than in HC (32). More importantly, serum valine levels in pMCI patients were negatively correlated with Tau and pTau levels in CSF and could be used to predict the risk of progression from MCI to AD (32). In a prospective study of eight cohorts, BCAAs were found to be negatively associated with dementia and the risk of AD (31). However, in another observational study, serum isoleucine levels were elevated in dementia, compared with HC (33). Similarly, elevated blood levels of BCAAs in patients with AD were reported in a small sample study cohort (17). In APP/PS1 mice model, there was an increased plasma BCAA level compared with wide-type mice (34). After high-fat feeding, the plasma BCAA level was significantly upregulated in the WT group, which was not observed in the APP/PS1 group (34). A high saturated fat/glycemic index diet significantly increased CSF BCAA levels in MCI patients but had no significant effect on the HC group (35). Combined with the results of this study, we speculate that these inconsistent results occurred in ordinary observational studies due to the limited sample size and the susceptibility of BCAAs to various confounding factors such as diet. The results of our study explain the causal relationship between AD and BCAAs from the root.

In 2017, Larsson and Markus also reported the results of a study using Mendelian randomization to explore the causal association of BCAAs with the risk of AD (36). The results showed that only higher isoleucine levels were associated with the risk of AD but not leucine and valine (36). Our study differs from other studies in several ways. In this study, the GWAS datasets related to BCAAs are selected with a larger sample size, containing 115,047 participants. From the perspective of methodology, Larsson and Markus (36) only used a single methodology to illustrate the effect of BCAAs on the risk of AD. In our study, IVW, MR-Egger, and weighted median are adopted in the analysis for Mendelian randomization, which provides an important guarantee for the reliability of our research results.

Branched-chain amino acids (BCAAs) are essential amino acids and account for one-third of the total amino acid content of the human body (30). After being absorbed by the gut, BCAAs are widely distributed in many organs for metabolism, mainly including the muscle, brain, liver, and adipose tissue (37). Among them, the muscle is the main metabolic place of BCAAs, accounting for approximately 50% (30, 38). In addition, the brain also absorbs a proportion of BCAAs in the blood (30). In the mammalian brain, BCAAs are involved in multiple functions, including neurotransmitter synthesis, protein synthesis, and energy production (39). In this study, AD patients are found to have reduced BCAA levels, which may be related to BCAA intake and metabolic processes. Circulating BCAAs are derived from diet, proteolysis, and gut microbiota (40). First, changes in gastrointestinal function have been observed in the AD mice model (41, 42). This may lead to reduced absorption of BCAAs from the diet in AD. Second, AD or MCI patients usually experience subclinical malnutrition (43). This resulted in reduced protein reserve and proteolysis levels in AD. Third, a series of studies in recent years have demonstrated the imbalance of gut microbiota in AD (12, 41). This may lead to changes in the abundance of gut microbiota related to BCAAs metabolism and ultimately reduce the level of circulating BCAAs. However, these are just some possible reasons why AD has reduced BCAA levels. Further studies are needed to clarify the specific mechanism. In addition, supplementing seven amino acids (leucine, phenylalanine, lysine hydrochloride, isoleucine, histidine hydrochloride, valine, and tryptophan) can improve cognitive, psychological, and social functions in middle-aged and older adults (44). Furthermore, supplementing L-norvaline (an isoform of valine) for 2 months can reverse cognitive decline and synaptic loss in triple-transgenic (3 × Tg) mice at the age of 4 months (45). Therefore, the supplementation of BCAAs may be one of the strategies to delay cognitive decline in AD patients.

## Conclusion

This bidirectional MR study indicates that AD is associated with decreased BCAA levels, which can serve as a marker for early diagnosis of AD. Future studies need to further clarify how AD leads to lower levels of BCAAs.

## Data availability statement

The original contributions presented in the study are included in the article/Supplementary material, further inquiries can be directed to the corresponding authors.

## Author contributions

H-dT and G-yD designed the study and prepared the manuscript. X-hQ and X-lL developed the methodology and analyzed the data. All authors discussed the results and approved the manuscript.

## Supplementary material

Supplementary Figure S1 Funnel plot of the causal effect between BCAAs level and the risk of AD. **(A)** Total BCAAs on AD, **(B)** valine on AD, **(C)** leucine on AD, **(D)** isoleucine on AD, **(E)** AD on total BCAAs, **(F)** AD on valine, **(G)** AD on leucine, **(H)** AD on isoleucine. Each instrumental SNP was represented by a black dot. SNPs, single-nucleotide polymorphisms; BCAAs, branched-chain amino acids; AD, Alzheimer's disease.

Supplementary Figure S2 Leave-one-out analysis of the causal effect between BCAAs level and the risk of AD. **(A)** Total BCAAs on AD, **(B)** valine on AD, **(C)** leucine on AD, **(D)** isoleucine on AD, **(E)** AD on total BCAAs, **(F)** AD on valine, **(G)** AD on leucine, **(H)** AD on isoleucine. BCAAs, branched-chain amino acids; AD, Alzheimer's disease.

Supplementary Table S1 Characteristics of selected SNPs for analysis of the causal effect between BCAA level and the risk of AD from IGAP.

Supplementary Table S2 F statistic in this study.
