# Supplementary figures and images for: Investigating the causal association between branched-chain amino acids and Alzheimer's disease: A bidirectional Mendelian randomized study
Source: Front Nutr. 2023 Mar 31;10:1103303. doi: 10.3389/fnut.2023.1103303 (PMC10102518; doi:10.3389/fnut.2023.1103303)

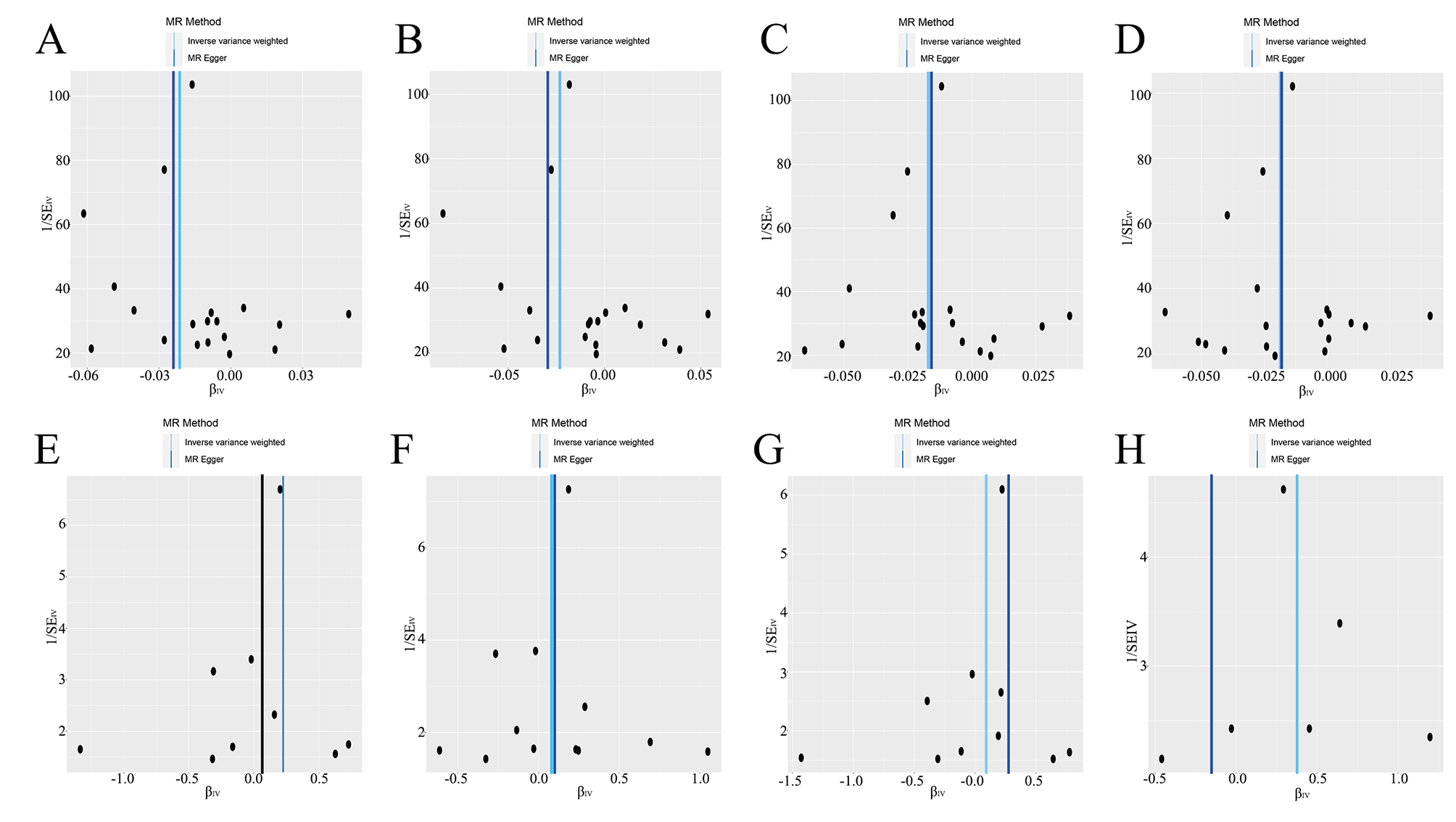

Supplement: Supplementary Figure S1 — Funnel plot of the causal effect between BCAAs level and the risk of AD. (A) Total BCAAs on AD, (B) valine on AD, (C) leucine on AD, (D) isoleucine on AD, (E) AD on total BCAAs, (F) AD on valine, (G) AD on leucine, (H) AD on isoleucine. Each instrumental SNP was represented by a black dot. SNPs, single-nucleotide polymorphisms; BCAAs, branched-chain amino acids; AD, Alzheimer's disease. [file Image_1.TIF]

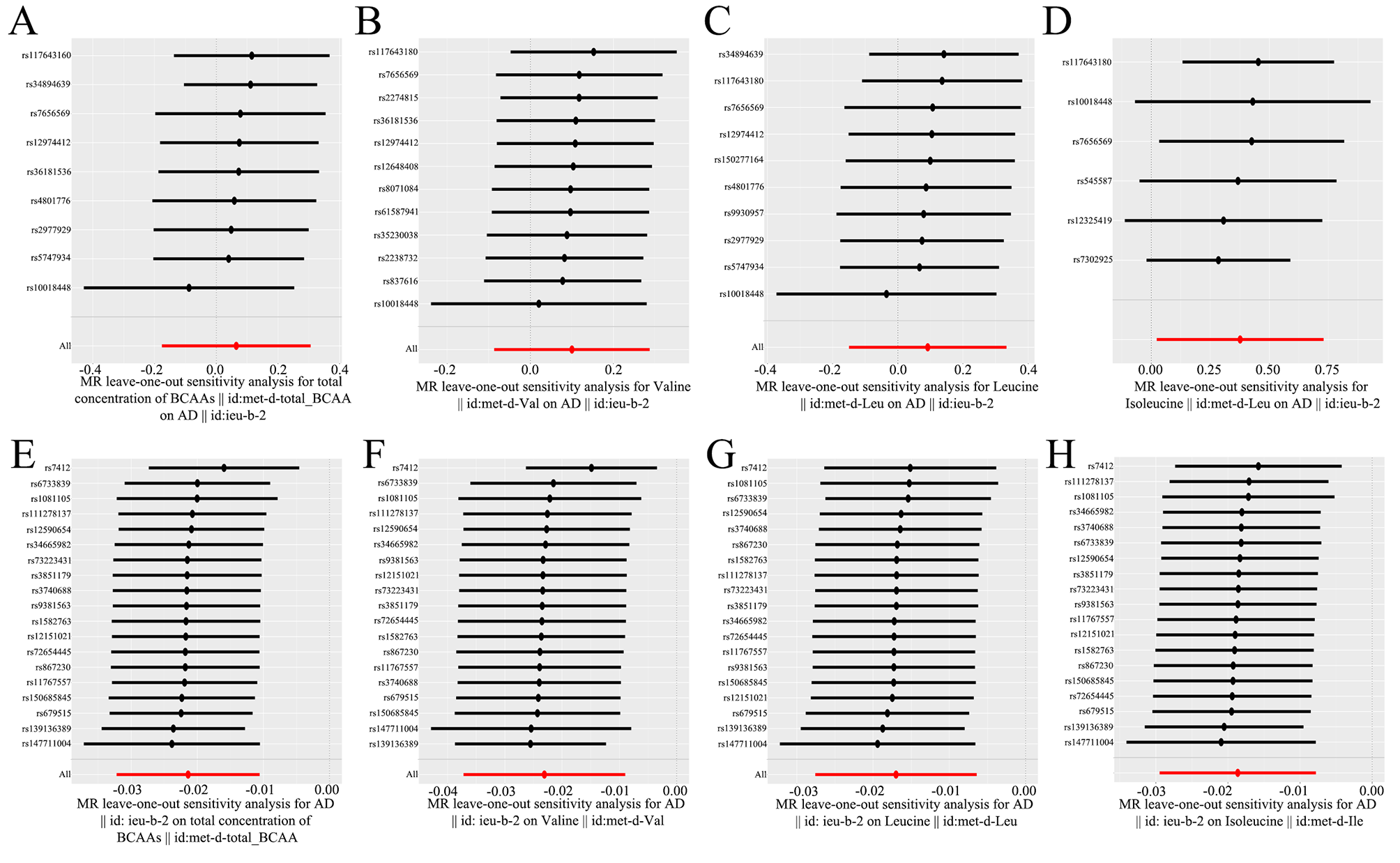

Supplement: Supplementary Figure S2 — Leave-one-out analysis of the causal effect between BCAAs level and the risk of AD. (A) Total BCAAs on AD, (B) valine on AD, (C) leucine on AD, (D) isoleucine on AD, (E) AD on total BCAAs, (F) AD on valine, (G) AD on leucine, (H) AD on isoleucine. BCAAs, branched-chain amino acids; AD, Alzheimer's disease. [file Image_2.TIF]
