# Supplementary material for: Investigating the causal association between branched-chain amino acids and Alzheimer's disease: A bidirectional Mendelian randomized study
Source: Front Nutr. 2023 Mar 31;10:1103303. doi: 10.3389/fnut.2023.1103303 (PMC10102518; doi:10.3389/fnut.2023.1103303)
Supplement: Supplementary Table S2 — F statistic in this study. [file Table_2.docx]

| Exposure | Outcome | F statistic |
| --- | --- | --- |
| Total BCAAs | AD  (IGAP) | 47.880 |
| Valine | AD  (IGAP) | 3.153 |
| Leucine | AD  (IGAP) | 37.091 |
| Isoleucine | AD  (IGAP) | 41.729 |
| AD  (IGAP) | Total BCAAs | 22.281 |
| AD  (IGAP) | Valine | 22.281 |
| AD  (IGAP) | Leucine | 22.281 |
| AD  (IGAP) | Isoleucine | 22.281 |
